# Highly purified hypochlorous acid water facilitates glucose metabolism and memory formation in type 2 diabetic mice associated with altered-gut microbiota

**DOI:** 10.1038/s41598-024-67129-z

**Published:** 2024-07-12

**Authors:** Kazuki Watanabe, Yusuke Maruyama, Risako Mikami, Keiji Komatsu, Kenji Kikuchi, Kunimoto Hotta, Toshikazu Yoshikawa, Kouetsu Ogasawara, Atsuhiko Hattori, Shinichi Arakawa

**Affiliations:** 1 Department of Immunobiology, Institute of Development, Aging and Cancer, Tohoku University, Sendai, Miyagi 980-8575 Japan; 2 Department of Biology, College of Liberal Arts and Sciences, Tokyo Medical and Dental University (TMDU), Ichikawa, Chiba 272-0827 Japan; 3 Department of Sport and Wellness, College of Sport and Wellness, Rikkyo University, Niiza, Saitama 352-8558 Japan; 4 Graduate School of Medical and Dental Sciences, Medical and Dental Science and Technology, Lifetime Oral Health Care Science, Tokyo Medical and Dental University (TMDU), Bunkyo-Ku, Tokyo, 113-8510 Japan; 5 Louis Pasteur Center for Medical Research, Tanaka Monzencho, 103-5, Sakyo-ku, Kyoto, 606-8225 Japan; 6 Department of Gastroenterology and Hepatology, Kyoto Prefectural University of Medicine, Kyoto, 602-8566 Japan; 7 Department of Oral Health Sciences, Faculty of Health Care Sciences, Takarazuka University of Medical Health, Nakatsu, 6-9-38, Kita-Ki, Osaka, 531-0071 Japan

**Keywords:** Cell biology, Neuroscience

## Abstract

Hypochlorous acid (HOCl) is an endogenous oxidant and chlorinating agent in mammals that is effective against a broad range of microorganisms. However, the effects of exogenous HOCl on biological processes have not been reported. In this study, the effects of highly purified slightly acidic hypochlorous acid water (HP-HAW) were investigated. After the safety of oral administration of HP-HAW was confirmed, the effects of HP-HAW on glucose homeostasis were assessed in mice. HP-HAW treatment significantly improved blood glucose levels in hyperglycemic condition. Based on the 16S rRNA sequencing, HP-HAW treatment significantly increased the diversity and changed the composition of gut microbiota by decreasing the abundance of genus *Romboutsia* in mice fed normal chow. In obese mice, HP-HAW administration tended to improve glucose tolerance. HP-HAW also attenuated memory impairments and changes *N*-methyl-d-aspartate (NMDA) receptor mRNA expression in obese mice. HP-HAW treatment suppressed *Il-6* mRNA expression in the hippocampus in type 2 diabetic mice. Overall, these results support HP-HAW as a potential therapeutic agent to improve or prevent glucose tolerance and memory decline via gut microbiota alteration.

## Introduction

Blood glucose regulation is one of the most important energy control systems in mammals. Blood glucose is required for normal metabolic processes, and poor glycemic control causes many chronic disorders, including diabetes mellitus (DM). DM, which is one of the most common metabolic disorders in the world, is characterized by hyperglycemia due to decreased insulin secretion or insulin resistance^1^. Complications, such as nephropathies, cardiovascular abnormalities, neuropathies, and cognitive impairments, cause high morbidity and mortality in patients with diabetes^2,3^. The prevalence of diabetes is increasing worldwide; the International DM Federation predicts that 592 million people worldwide will suffer from DM by 2035^4^. Thus, the development of effective strategies for the prevention and management of DM and its complications is essential.

Reactive oxygen species (ROS) are molecular oxygen derivatives and free radicals with redox activity^5^. ROS include free radicals, such as superoxide, hydroxyl radical, and peroxyl radical, and non-radical molecular oxygen species, such as hydrogen peroxide, singlet oxygen, and hypochlorous acid (HOCl)^6^. HOCl, which is endogenously produced in mammals, is a strong oxidant and chlorinating agent that is efficient in killing a broad range of microorganisms, including bacteria and pathogenic fungi^7^. HOCl also promotes human endothelial cell survival via enhancing heme oxygenase-1 gene expression^8^. HOCl is widely used to prevent nosocomial infections in hospitals, including antibacterial intracanal irrigation, oral maxillofacial surgery, and mouthwash. The in vivo safety profile of HOCl has been reported for various animal models^9^. However, little information is available concerning the safety and effects of the oral administration of highly purified slightly acidic hypochlorous acid water (HP-HAW). A wide variety of hypochlorous acid water products is available worldwide, including HP-HAW. HP-HAW is stable, with a HClO decomposition rate of about 5% per year at 5 °C.

After establishing the safety of oral HP-HAW treatment, we investigated the effects of HP-HAW on biological processes, including glucose homeostasis, in mice. In addition, we investigated the impact of HP-HAW administration on gut microbiota, memory formation, and inflammation-related genes in the hippocampus in type 2 diabetic mice.

## Results

### Effects of HP-HAW on body weight and glucose tolerance in mice fed normal chow

**Figure 1:**
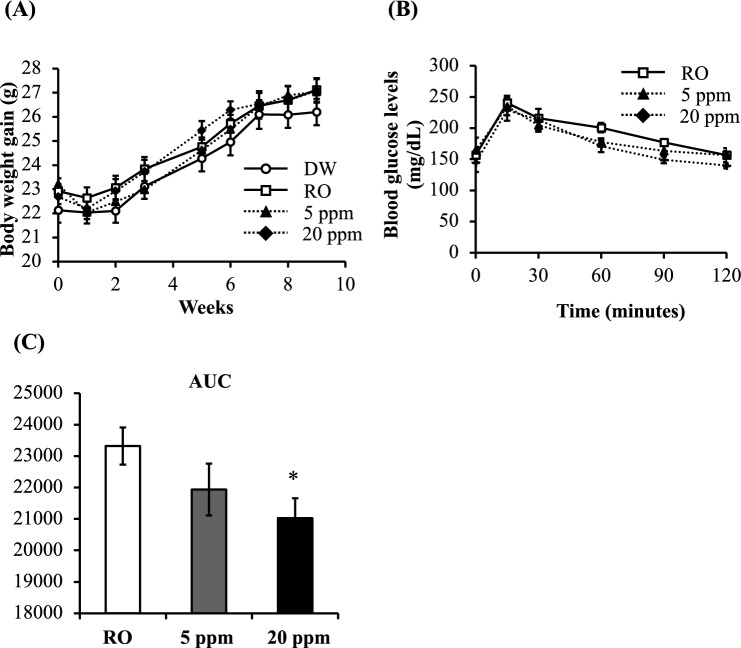
Comparison of body weight and glucose tolerance in mice fed a normal chow diet and given distilled water (DW), reverse osmosis (RO) water, or highly purified slightly acidic hypochlorous acid water (HP-HAW, 5 ppm or 20 ppm). (**A**) Body weight gain after DW, RO water, or HP-HAW (5 ppm and 20 ppm) treatment. (**B**) Changes in plasma glucose levels induced by hyperglycemic conditions. Glucose (1 g/kg body weight) was administered intraperitoneally. Blood samples were collected before and 15, 30, 60, 90, and 120 min after glucose administration, and plasma glucose levels were measured. (**C**) The area under the curve. *P < 0.05 vs. the RO treated group.

No significant differences in body weight were detected between the distilled water (DW), reverse osmosis (RO) water, and HP-HAW (5 and 20 ppm) mouse treatment groups fed normal chow (Fig. 1A). After 10 weeks of treatment, glucose tolerance improved in mice in the HP-HAW group compared with mice in the control mice (Fig. 1B,C).

### HP-HAW alters the diversity and composition of the gut microbiota in mice fed normal chow

**Figure 2:**
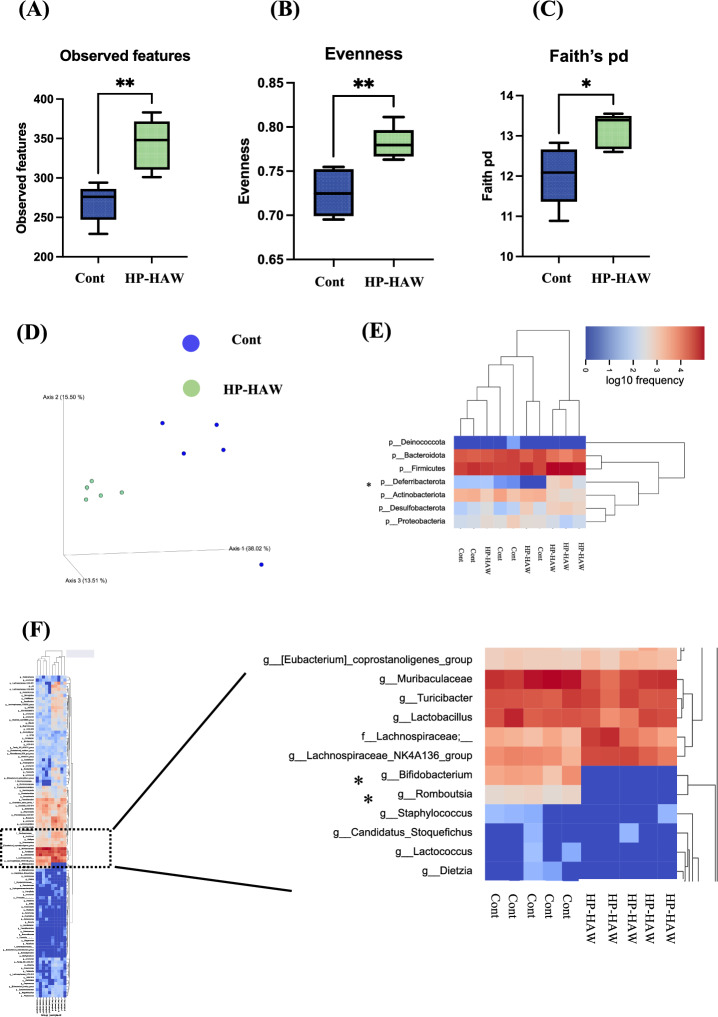
Evaluation of gut microbial biodiversity based on 16S rRNA gene sequences in mice fed a normal chow diet and treated with reverse osmosis (RO) or highly purified slightly acidic hypochlorous acid water (HP-HAW, 20 ppm). (**A**) Observed features, (**B**) Evenness, and (**C**) Faith’s phylogenic diversity (pd). (**D**) Principal coordinate analysis. Dendrogram and heatmap based on read abundance at the (**E**) phylum and (**F**) genus levels. *P < 0.05 and **P < 0.01 vs. the RO water group.

Observed features, evenness, and Faith’s phylogenic diversity (pd) differed significantly between the control group (RO water treatment group) and the HP-HAW treatment group (Fig. 2A–C), suggesting that HP-HAW significantly improved the diversity of gut microbiota. Principal coordinate analysis (PCoA) revealed that the microbiome compositions differed dramatically between the control and HP-HAW groups (Fig. 2D).

### HP-HAW alters the relative abundance of gut microbiota in mice fed normal chow

As shown in Fig. 2E, the phylum-level analysis demonstrated that HP-HAW significantly increased the relative abundance of *Deferribacterota* in mice fed normal chow. The genus-level analysis demonstrated that the relative abundance of *Bifidobacterium* and *Romboutsia* decreased in mice treated with HP-HAW compared with mice treated with RO water (Fig. 2F).

### Effects of HP-HAW on body weight and glucose tolerance in mice fed a high-fat diet

**Figure 3:**
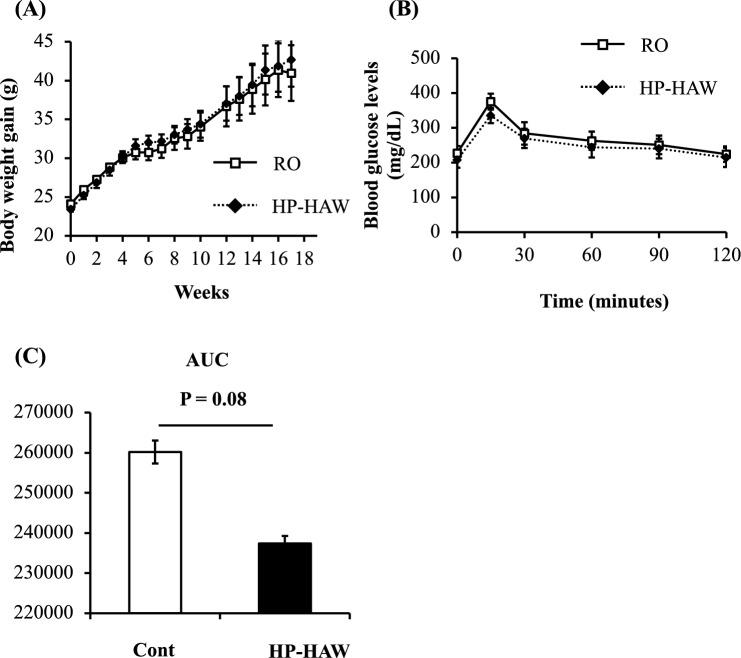
Comparison of body weight and glucose tolerance in mice fed a high-fat diet and treated with reverse osmosis (RO) or highly purified slightly acidic hypochlorous acid water (HP-HAW, 20 ppm). (**A**) Body weight gain after treatment with RO water or HP-HAW (20 ppm). (**B**) Plasma glucose levels under induced hyperglycemic conditions. Blood samples were collected before and 15, 30, 60, 90, and 120 min after intraperitoneal administration of glucose (1 g/kg body weight), and plasma glucose levels were measured. (**C**) The area under the curve.

No significant differences in body weights were detected between the control (RO water treatment) and HP-HAW-treated (20 ppm) mice fed a high-fat diet (Fig. 3A). After 10 weeks, glucose tolerance tended to improve in mice treated with HP-HAW compared with mice treated with RO water (Cont) (Fig. 3B,C).

### Effects of HP-HAW on long-term memory performance and NMDA receptor gene expression in the hippocampus

**Figure 4:**
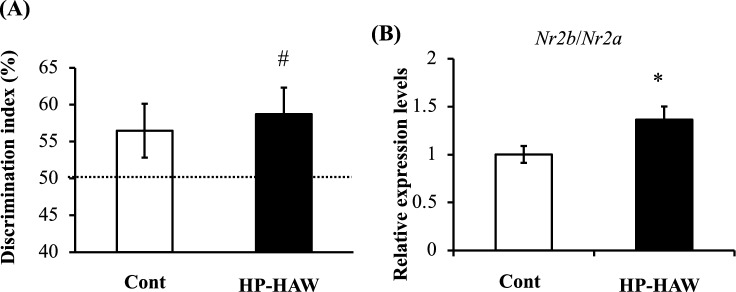
Long-term object memory and NMDA receptor gene expression levels in mice fed a normal chow diet and treated with reverse osmosis (RO) or highly purified slightly acidic hypochlorous acid water (HP-HAW, 20 ppm). (**A**) The discrimination index (DI) was calculated as the percentage of time spent exploring the novel object divided by the total time spent exploring both objects. (**B**) *Nr2b*/*Nr2a* mRNA expression levels in the hippocampus of mice treated with RO (Cont) or HP-HAW. Data are presented as means ± standard errors. ^#^*P* < 0.05 vs. chance performance (50%). *P < 0.05 vs. RO treated group.

The effects of HP-HAW on memory formation were assessed using the novel object recognition (NOR) test. The discrimination index (DI) in the HP-HAW group was significantly higher than chance performance. In contrast, the DI for the control group was not significantly different from chance performance (50%) (Fig. 4a). The mRNA levels for the NMDA receptors *Nr2b* and *Nr2a* were measured in the hippocampus of mice. The *Nr2b*/*Nr2a* ratio in the hippocampus from the HP-HAW group was significantly higher than the *Nr2b*/*Nr2a* ratio in control group (Fig. 4b).

### HP-HAW suppressed the expression of inflammatory mediators in the hippocampus of mice

**Figure 5:**
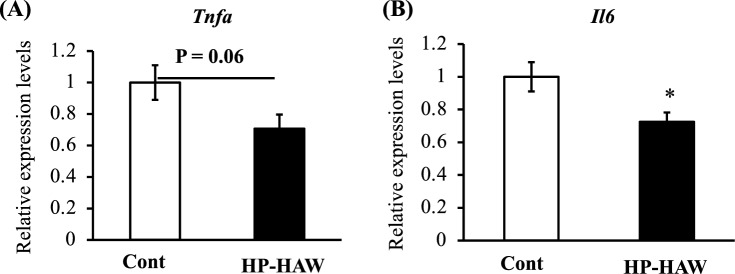
Inflammatory-related gene expression in the hippocampus of mice fed a high-fat diet and treated with reverse osmosis (RO) or highly purified slightly acidic hypochlorous acid water (HP-HAW, 20 ppm). (**A**) Tumor necrosis factor alpha (*Tnfa*) and (**B**) interleukin-6 (*Il6*) mRNA expressions in mice treated with RO water or HP-HAW. *P < 0.05 vs. RO group.

The mRNA levels for inflammatory mediators, including tumor necrosis factor alpha (*Tnfa*) and interleukin-6 (*Il6*), in the hippocampi of mice were measured. *Tnfa* mRNA levels tended to be lower and *Il6* mRNA levels were significantly lower in the HP-HAW group compared with the levels in the control group (Fig. 5a,b).

### Evaluation of gut microbiome composition based on 16S rRNA gene sequences in mice fed a high-fat diet

**Figure 6:**
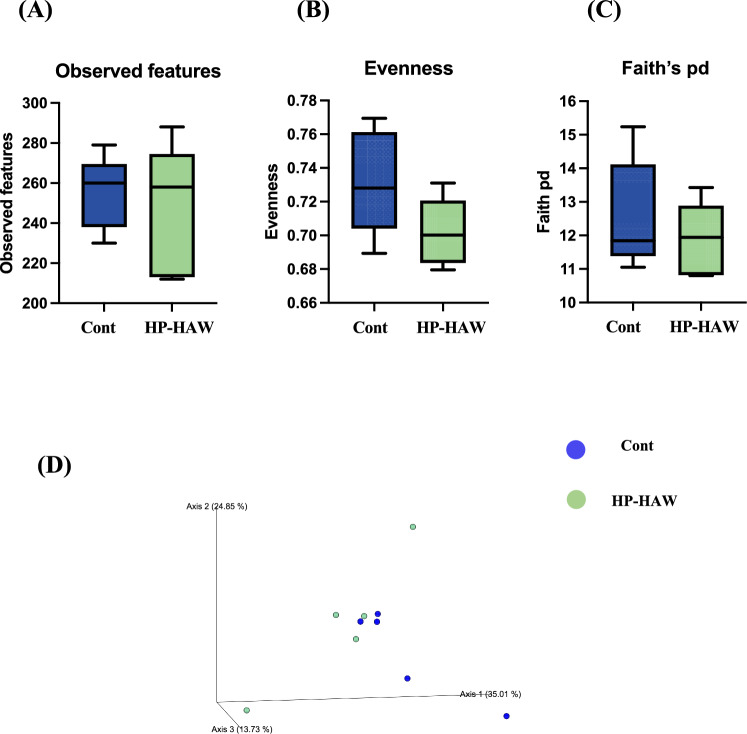
Evaluation of gut microbial biodiversity based on 16S rRNA gene sequences in mice fed a high-fat diet and treated with RO (Cont) or highly purified slightly acidic hypochlorous acid water (HP-HAW, 20 ppm). (**A**) Observed features, (**B**) Evenness, and (**C**) Faith’s phylogenic diversity (pd), and (**D**) Principal component analysis.

No significant differences in the observed features (Fig. 6A), evenness (Fig. 6B), or Faith’s pd (Fig. 6C) indices were detected between the control and HP-HAW mice. However, PCoA revealed different microbiome compositions in the control and HPPAW mice (Fig. 6D).

## Discussion

The safety of HP-HAW was determined first. Several biochemical parameters, including albumin, BUN, total cholesterol, AST, and ALT did not change in response to HP-HAW treatment (Supplementary Table S1), demonstrating the safety of HP-HAW. Furthermore, our results showed that the treatment of HP-HAW decreased the blood TNF-α levels. After confirming the safety of HP-HAW, the effects of RO water (control) and HP-HAW were compared. HP-HAW significantly attenuated blood glucose increases, increased the diversity of gut microbiota, and improved long-term memory performance by inhibiting inflammatory cytokine gene expression in the hippocampus. The intestinal microbial community, the gut microbiota, lives in a mutualistic relationship with its host and produces vitamins and the other metabolites that are beneficial for host physiology. The gut microbiome is a complex community of microorganisms in the digestive tract of humans and animals. The gut microbiota perform significant functions, including enhancing the immune system^10^, contributing to metabolism^11^, and modifying insulin resistance and secretion^12^. However, several diseases such as cardiovascular disease^13^, autoimmune disease^14^, and obesity and diabetes^15^ are related the gut microbiota. Microbiota diversity makers (observed features, evenness, and Faith’s pd) were increased by HP-HAW treatment in mice fed normal chow, attenuating the increased blood glucose levels. At the genus level, *Romboutsia* was increased in the HP-HAW group. In a previous study, the genus *Romboutsia* was significantly increased in patients with type 2 diabetes^16^. Thus, HP-HAW may affect glucose homeostasis by decreasing the abundance of *Romboutsia* in the gut.

This study has several limitations in terms of the analysis of the gastrointestinal tract. First, we could not observe the effect of HP-HAW on the pathology of the gastrointestinal tract. HP-HAW may cause some changes in the gastrointestinal tract, for example, histological morphology. Second, HP-HAW might affect intestinal bacteria. The mucus layer of the intestine acts as a physical barrier by maintaining bacteria in symbiosis with the host and preventing bacterial infiltration into the epithelium. The large intestinal epithelium is covered by two mucus layers^17^. The luminal bacteria could penetrate the mucous layer and exert effects on the intestinal tissue. Despite being a barrier against the gut microbiota, intestinal mucus requires the presence of bacteria and the outer mucus layer is rich in gut bacteria^18^. Mucins, one of the major components of the mucosal layer, are a source of nutrients for intestinal bacteria because they are composed of amino acids and oligosaccharides. The inner layer is devoid of bacteria in healthy individuals, whereas the outer mucus layer is colonized with an abundance of commensal bacteria, particularly mucin-degrading bacteria, such as *Akkermansia muciniphila*, *Bifidobacterium bifidum*, and *Ruminococcus gnavus*^19,20^. Among them, *A. muciniphila* has a substantial impact on host physiology and microbiome composition^21^. In the gut, the abundance of *A. muciniphila* is negatively correlated with numerous diseases, including inflammatory bowel diseases^22^, obesity^23^, and diabetes^24^. Therefore, HP-HAW may affect intestinal bacteria, including *A. muciniphila* and gut microbiota. Further analyses, such as fecal transplantation experiments, are required to determine the effects of HP-HAW on the gut microbiota,

In mice fed a high-fat diet, HP-HAW treatment tended to decrease blood glucose levels, which were measured using the glucose tolerance test, compared with the control group. No significant differences in gut microbiota diversity were detected between the HP-HAW treatment and control groups. Significant changes in taxonomic diversity may be observed if the HP-HAW dosing period is altered. Oral microbiota can translocate to the intestinal mucosa and affect the gut microbiota. For example, we reported that dysbiosis of oral microbiota caused dysbiosis of gut microbiota^25^. Therefore, HP-HAW may affect gut microbiota by changing oral microbiota.

Interestingly, HP-HAW treatment facilitated long-term memory in mice fed a high-fat diet. The NMDA receptor is crucial to the development of neuronal differentiation and synaptogenesis^26^. NR2B expression levels are higher in the neonate than in older age groups. NR2A expression levels are constant, leading to an age-related decrease in the NR2B/NR2A ratio^27^. A high-fat diet causes memory dysfunction and impairs hippocampus neurogenesis^28,29^. HP-HAW suppressed the expression levels of inflammation-related genes in the hippocampus, suggesting that HP-HAW improves memory formation by attenuating hippocampal inflammation and protecting neurogenesis. Dementia is one of the most severe complications of diabetes, and HP-HAW might be a potential agent to improve memory formation in individuals with diabetes.

Cellular ROS are generated endogenously in the process of mitochondrial oxidative phosphorylation or arise from interactions with exogenous sources such as xenobiotic compounds^30^. Myeloperoxidase (MPO) is an abundant constituent of neutrophil extracellular traps (NETs). NETs are unique extracellular structures composed of uncondensed chromatin decorated with nuclear and cytosolic proteins with antimicrobial activity. These structures are formed by neutrophils in response to pathogens. MPO activity decreases in patients with diabetes^31^, and exogenous HOCl may be a potential drug for glucose regulation. Although the precise effects of HP-HAW on oral/gut microbiota must await further study, the application of HP-HAW to the oral cavity may be important for symbiosis in oral/gut microbiota. In conclusion, this study revealed that HP-HAW is a potential therapeutic agent to improve or prevent glucose tolerance and memory decline by altering gut microbiota.

## Materials and methods

### Highly purified slightly acidic Hypochlorous acid water (HP-HAW)

A novel production system developed by Nipro Co. was used (International application number: WO 2023/090447 Al and WO 2021/235554 Al). A high concentration of pure NaCl was dissolved in pure water using a three-chambered electrolyzer (AW03α, Osaka Japan). The resulting hypochlorous acid water was filtered through an RO membrane to remove ions such as Na^+^. The resulting solution exhibited the following properties: HClO 18–21 mg/L, pH 6.5–7.0, electric conductivity 9–15 μS/cm, Na^+^ 2–5 mg/L, and Cl^-^ 4–10 mg/L. The solution was highly stable due to its extremely low electric conductivity; the decomposition rate of HClO was about 5% per year at 5 °C.

### Ethical treatment of animals

This study was conducted in accordance with the recommendations of the ethical guidelines of Tokyo Medical and Dental University (TMDU). All experimental protocols were approved by the Animal Welfare Committee of TMDU (permit number: A2021-276A). Additionally, the protocols were in accordance with ARRIVE guidelines 2.0^32^. All experiments were performed in a manner that minimized pain and discomfort.

### Animals

C57BL/6J mice (7 weeks old; Sankyo Laboratory, Tokyo, Japan) were fed a normal chow diet or high-fat diet 32 (CLEA Japan, Tokyo, Japan) for 17 weeks. High-fat diet 32 is composed of 24.5% milk casein, 5.0% albumin powder, 0.43% l-cystine, 15.88% powdered beef tallow, 20.0% safflower oil, 5.5% crystalline cellulose, 8.25% maltodextrin, 6.928% lactose, 6.75% sucrose, 1.4% American Institute of Nutrition (AIN)-93 vitamin mix, 5.0% AIN-93G mineral mix, 0.36% choline hydrogen, and 0.002% tertiary butyl hydroquinone. Mice received DW, RO water, or HP-HAW (5 ppm and 20 ppm diluted in RO water) as drinking water for 17 weeks. The normal diet experiment included 5 individuals per group and the high-fat diet experiment included 8–9 individuals per group. HP-HAW was prepared twice a week and stored in a bottle with a blackout cover to prevent light-induced degradation. Glucose tolerance and was measured as previously described^25^. After fasting for 6 h, mice were fed glucose by oral gavage (1 g/kg body weight) at 10 weeks. Glucose concentration was determined with a glucose meter (Nipro Stat Strip XP; Nipro, Osaka, Japan). The area under the curve (AUC) (0–120 min) was calculated for each treatment group.

### 16S rRNA gene sequencing and illumina sequence data processing

DNA was extracted from mouse feces and a multiplexed amplicon library (16S rDNA V3–V4 region) was generated. Sequences were obtained, as previously described^25^, using a MiSeq platform (Illumina, San Diego, USA) to obtain 2 × 250 base-pair paired-end reads. The sequence data were processed and analyzed using QIIME2 (version 2021.2). Taxonomic classification of amplicon sequence variants was performed using QIIME feature-classifier classify-sklearn, based on SILVA 138 at 99% sequence similarity. Diversity metrics were calculated using the core diversity plugin within QIIME2. Feature level alpha diversity indices, including observed features, evenness, and Faith’s pd index, were calculated to estimate the microbial diversity within an individual sample. The beta diversity was visualized via PCoA. Differentially abundant taxa were identified with Analysis of Composition of Microbiomes (ANCOM) R package^33^.

### Novel object recognition test (NOR)

NOR tests were performed, as previously described^34^. Mice tested after 17 weeks of treatment. Mice were individually habituated to the empty arena for 5 min daily for 3 days. In the training phase, animals performed one acquisition trial (5 min) where they were allowed to freely explore two identical objects symmetrically placed in the arena 6 cm from the wall and 6 cm from each other. Mice were tested 24 h later. One of the familiar objects was replaced with a novel object at the same location and the mice were given 3 min to freely explore. Object novelty and location were counterbalanced within experimental groups to eliminate potential biases caused by preferences for particular objects or locations. The time spent exploring the objects was quantified by a blinded trained observer using the recordings. The DI was calculated as follows: % time spent exploring the novel object/total time spent exploring both objects during the test phase. Object recognition was defined as a DI significantly higher than chance performance (50%)^35,36^.

### RNA preparation and complementary DNA synthesis

Total RNA was extracted from the hippocampus of mice using a NucleoSpin^®^ RNA kit (TaKaRa Bio, Shiga, Japan) according to the manufacturer’s instructions. Total RNA (1 μg) was reverse-transcribed to cDNA using PrimeScript Reverse Transcriptase and oligo dT primers (Takara Bio).

### Quantitative real-time PCR

Quantitative real-time PCR (qPCR) was performed using primers for inflammation-related genes and genes related to memory formation (Table 1). β-actin was used as an internal control. No amplified products were found when reverse transcriptase was omitted from the reaction as a negative control. Amplification was performed using SYBR Ex *Taq* DNA Polymerase (Takara Bio) on a Mx3000P qPCR System (Agilent Technologies, Santa Clara, CA, USA). The qPCR protocol consisted of 40 cycles of denaturation at 95 °C for 10 s and annealing/extension at 65 °C for 40 s, followed by melt curve analysis.

**Table 1:** Primers used for quantitative real-time PCR.

Gene name	Forward primer	Reverse primer
*β-actin*	TACTGCTCTGGCTCCTAGCA	CGGACTCATCGTACTCCTGC
*Nr2b*	GCCATGAACGAGACTGACCC	GCTTCCTGGTCCGTGTCATC
*Nr2a*	TGATGAACCGCACTGACCCTA	TGGGGATGAAAGTCTGTGAGG
*Tnfa*	GCAACCCTTATTCTCGCTCA	TCCACACTCTCCTCCACCTT
*Il6*	GATACCACTCCCAACAGACCT	ATTTCCACGATTTCCCAGA

### Measurement of biochemical parameters and blood inflammation parameters

Plasma was separated from blood samples by centrifugation (3000 rpm, 10 min).

Biochemical parameters including triglyceride (TG), total cholesterol (T-CHO), aspartate aminotransferase (AST), and alanine aminotransferase (ALT) were measured by a local laboratory specialized in clinical analyses (Oriental Yeast Co., Ltd., Shizuoka, Japan). Plasma TNF-α and CRP levels were determined by an enzyme-linked immunosorbent assay kit (ELISA). All procedures were performed according to the manufacturer’s instructions (TNF-α ELISA kit and CRP ELISA Kit; Proteintech, Rosemont, illinois, USA).

### Statistical analysis

Data are presented as means ± standard error. The means of more than two groups were compared using analysis of variance (ANOVA). Multiple comparisons were evaluated using Dunnett’s multiple comparison tests. Differences between the treatment and control groups were assessed using Student’s *t*-tests. Assessment of significantly different taxa between different treatment groups was performed using the ANCOM program in QIIME 2. *P* values < 0.05 was considered statistically significant.

### Supplementary Information

Supplementary Table S1.

## Data Availability

The original contributions presented in the study are included in the article/supplementary material. The 16S rRNA gene sequence data are deposited at DNA Data Bank of Japan (DDBJ; https://www.ddbj.nig.ac.jp/index-e.html) Sequence Read Archive (DRA), under the accession number DRA017639. Further inquiries can be directed to the corresponding author.
