# Supplementary material for: Highly purified hypochlorous acid water facilitates glucose metabolism and memory formation in type 2 diabetic mice associated with altered-gut microbiota
Source: Sci Rep. 2024 Jul 12;14:16107. doi: 10.1038/s41598-024-67129-z (PMC11245604; doi:10.1038/s41598-024-67129-z)
Supplement: Supplementary file 1 — Supplementary Table S1. [file 41598_2024_67129_MOESM1_ESM.pdf]

Supplementary_table_S1: Biochemical parameters and blood inflammation markers in control mice and HP-HAW treated mice.

	All mice	Control	HP-HAW
**TP (g/dL)**	5.21 ± 0.06	5.3 ± 0.04	5.1 ± 0.12
**ALB (g/dL)**	3.26 ± 0.05	3.28 ± 0.06	3.23 ± 0.1
**BUN (mg/dL)**	23.83 ± 0.69	23.26 ± 1.18	24.55 ± 0.48
**CRE (mg/dL)**	0.09 ± 0	0.1 ± 0.01	0.09 ± 0
**Na (mEq/L)**	149.67 ± 0.5	149.2 ± 0.86	150.25 ± 0.25
**Cl (mEq/L)**	111.44 ± 0.63	110.6 ± 0.87	112.5 ± 0.65
**IP (mg/dL)**	6.5 ± 0.2	6.38 ± 0.3	6.65 ± 0.25
**AST (IU/L)**	119.22 ± 12.78	103.8 ± 12.29	138.5 ± 22.48
**ALT (IU/L)**	28 ± 2.8	23.4 ± 1.29	33.75 ± 4.96
**T-CHO (mg/dL)**	71.78 ± 3.37	73.6 ± 4.66	69.5 ± 5.36
**TG (mg/dL)**	34.89 ± 2.72	35 ± 4.53	34.75 ± 3.2
**HDL-C (mg/dL)**	40.78 ± 2.54	42.6 ± 2.64	38.5 ± 4.91
**TNF-α (pg/mL)**	19.99 ± 1.56	23.54 ± 1.72	16.44 ± 1.29*
**CRP (pg/mL)**	12.44 ± 1.47	13.91 ± 2.06	10.96 ± 2.10

Note: Values are mean ± SE.

*$P < 0.05$ compared to control.
